# Fabrication of Fish Scale-Based Gelatin Methacryloyl for 3D Bioprinting Application

**DOI:** 10.3390/polym16030418

**Published:** 2024-02-01

**Authors:** Kitipong Pasanaphong, Danai Pukasamsombut, Sani Boonyagul, Sukanya Pengpanich, Tulyapruek Tawonsawatruk, Danuphat Wilairatanarporn, Kittisak Jantanasakulwong, Pornchai Rachtanapun, Ruedee Hemstapat, Sutee Wangtueai, Nuttapol Tanadchangsaeng

**Affiliations:** 1 College of Biomedical Engineering, Rangsit University, Lak-Hok 12000, Pathumthani, Thailand; kitipong.pasanaphong@gmail.com (K.P.); danai.pu@hotmail.com (D.P.); sani@rsu.ac.th (S.B.); sukanya.pen@ncr.nstda.or.th (S.P.); danuphat.w@hotmail.com (D.W.); 2 National Center for Genetic Engineering and Biotechnology (BIOTEC), National Science and Technology Development Agency, Khlong Nueng 12120, Pathumthani, Thailand; 3 Department of Orthopaedics, Faculty of Medicine Ramathibodi Hospital, Mahidol University, Ratchathewi, Bangkok 10400, Thailand; tulyapruek.tao@mahidol.ac.th; 4 Division of Packaging Technology, School of Agro-Industry, Faculty of Agro-Industry, Chiang Mai University, Mae Hia, Muang, Chiang-Mai 50100, Thailand; kittisak.jan@cmu.ac.th (K.J.); pornchai.r@cmu.ac.th (P.R.); 5 Department of Pharmacology, Faculty of Science, Mahidol University, Ratchathewi, Bangkok 10400, Thailand; ruedee.hem@mahidol.ac.th; 6 College of Maritime Studies and Management, Chiang Mai University, Tha Chin, Muang, Samut Sakhon 74000, Thailand

**Keywords:** bioink, 3D bioprinting, fish scale gelatin, hydrogel scaffold, cell proliferation

## Abstract

Gelatin methacryloyl (GelMA) is an ideal bioink that is commonly used in bioprinting. GelMA is primarily acquired from mammalian sources; however, the required amount makes the market price extremely high. Since garbage overflow is currently a global issue, we hypothesized that fish scales left over from the seafood industry could be used to synthesize GelMA. Clinically, the utilization of fish products is more advantageous than those derived from mammals as they lower the possibility of disease transmission from mammals to humans and are permissible for practitioners of all major religions. In this study, we used gelatin extracted from fish scales and conventional GelMA synthesis methods to synthesize GelMA, then tested it at different concentrations in order to evaluated and compared the mechanical properties and cell responses. The fish scale GelMA had a printing accuracy of 97%, a swelling ratio of 482%, and a compressive strength of about 85 kPa at a 10% *w*/*v* GelMA concentration. Keratinocyte cells (HaCaT cells) were bioprinted with the GelMA bioink to assess cell viability and proliferation. After 72 h of culture, the number of cells increased by almost three-fold compared to 24 h, as indicated by many fluorescent cell nuclei. Based on this finding, it is possible to use fish scale GelMA bioink as a scaffold to support and enhance cell viability and proliferation. Therefore, we conclude that fish scale-based GelMA has the potential to be used as an alternative biomaterial for a wide range of biomedical applications.

## 1. Introduction

In recent years, the promise and potential of 3D bioprinting have shed light on the intersection of biomedical research and engineering. The technique of 3D bioprinting combines the complexity of biology with the accuracy of printing technologies. In biomedical applications like tissue engineering, regenerative medicine, and drug delivery, 3D bioprinting has emerged as a transformative technology [1]. Three-dimensional bioprinting techniques are categorized by the American Society for Testing and Materials (ASTM) into multiple groups. The three most common types are extrusion-based, jetting-based, and Vat photopolymerization-based bioprinting. The following bioprinting ink specifications apply to each technique: bioink used in extrusion techniques needs to have a suitable viscosity to keep its shape after extrusion, but it also needs to be liquid enough to allow for smooth extrusion and show shear-thinning behavior to help with extrusion and provide structural stability right away. Low-viscosity bioink is required for jetting in order to form tiny droplets without clogging the nozzle. Lastly, to facilitate crosslinking during VAT photopolymerization, the bioink needs to include a photo-initiator that reacts to light exposure and possesses the proper rheological characteristics to print with high efficiency.

The selection and functionality of bioink materials serve not only as passive carriers, but also as dynamic facilitators of cellular growth, organization, and function, and they are essential to realizing this visionary transformation [2]. Gelatin methacryloyl (GelMA) has established itself as a material of choice due to its biocompatibility, modifiable mechanical properties, and compatibility with a wide range of cell types [3]. GelMA’s precursor, gelatin, is mainly derived from mammalian sources, which raises ethical concerns, supply chain issues, the possibility of disease transmission, and cultural resistance [4]. This intricate web of obstacles highlights the need for a viable, less contentious, and at least as effective alternative to the status quo.

The fish processing industry is one of the major food industries, mainly producing fish mince or surimi products. Fish mince processing processes commonly generate approximately 50–70% byproducts, containing fish head, skin, scale, bone, and viscera. These underutilized resources are rich in protein and are utilized to obtain various high-value products. Fish scales and skins are primary collagenous sources that could be extracted to obtain fish collagen or gelatin for applications as biopolymers with good properties [5]. The use of biocompatible polymer products in a new form of GelMA may be widely applied and can help create high-value products of fish gelatin. In addition, the ethical and cultural concerns associated with mammalian sources could be avoided, allowing for a broader adoption of the resulting material. Furthermore, in an era dominated by sustainability imperatives, the reuse of fish scales aligns precisely with global waste reduction and resource optimization objectives [6]. Despite the attractiveness of the proposition, its realization is not a straightforward task. The utilization of fish scale gelatin into a refined, print-ready GelMA requires extensive study, intricate chemical engineering, and exhaustive characterizations to ensure the biocompatibility and efficacy of the bioink.

This study initiates an investigation into this transition. Using a combination of experimental methods, chemical engineering principles, and biomedical evaluations, we intend to elucidate the potential of fabricating GelMA from fish scales for 3D bioprinting applications. This expedition will investigate the biochemistry of fish scales, the complex chemical processes necessary for their transformation into GelMA, and the bioprinting performance of the resulting material. This research represents a broader philosophical shift in how we perceive and utilize waste, aiming to catalyze a paradigm shift in bioink procurement and expand the realm of the possibilities of 3D bioprinting to provide a fresh perspective on regenerative medicine and bioprinting.

## 2. Materials and Methods

### 2.1. Synthesis of GelMA Bioink

Fish scale gelatin is a type B gelatin extracted from lizardfish (*Saurida* spp.) scale using alkaline pretreatment and a hot water extraction method, as previously described [7]. This gelatin had 230 ± 2 g gel strength and contained 90 ± 0.5% crude protein. Methacrylic anhydride was obtained from Sigma Aldrich (St. Louis, MO, USA), phosphate-buffered saline was obtained from Millipore (Burlington, MA, USA), and 1-[4-(2-hydroxyethoxy)-phenyl]-2-hydroxy-2-methyl-1-propanone (Irgacure 2959) was obtained from Sigma-Aldrich. 

The conventional method [8] synthesized GelMA fish scales by substituting methacryloyl on the amine and hydroxyl amino groups. Briefly, at 60 °C, phosphate-buffered saline (PBS 1X, pH = 7.4) was used to dissolve fish scale gelatin. The gelatin solution was then given a dropwise addition of methacrylic anhydride at a rate of 1 mL/min, and the mixture was stirred for 30 min before being diluted with phosphate-buffered saline (PBS 5X, pH = 7.4) to stop the reaction. A dialysis bag (12–14 kDa molecular weight limit) was used to remove excess methacrylic anhydride that did not react, as well as other byproducts that might be dangerous to cells, from the mixed solution after it had been diluted with PBS 5x. Fresh deionized water was added to the dialysis solution every day for a week. After the reaction products were freeze-dried, a foam-like white solid resulted.

GelMA powder was dissolved in phosphate-buffered saline (PBS 1X, pH = 7.4) at 60 °C to produce GelMA hydrogels. Sterile material was put through a 0.2-micron filter, and an Irgacure 2959 photoinitiator was then added and crosslinked by 365 nm wavelength UV irradiation with an intensity of 10 mW/cm^2^ for 10 min.

### 2.2. NMR Characterization and Degree of Substitution Analysis

The ^1^H nuclear magnetic resonance spectrometer (proton NMR) was used to confirm the degree of substitution (DS) of free amino groups on gelatin by methacrylate groups, as well as to determine the degree of substitution of methacrylamide-modified gelatin. GelMa was dissolved at room temperature in deuterium oxide (D_2_O, for example, 10% *w*/*v*) and analyzed using ^1^H NMR spectroscopy (Varian Unity Inova 500 125 MHz). The peak area ratio of modified amino groups to main amino groups [8,9] can be calculated to determine the degree of substitution of methacrylamide-modified gelatin. In general, the degree of substitution calculation formula (Equation (1)) is as follows [10]:

DS = *S′*/*S* (1)

where *S′* represents the average integral area of modified amino groups (C=C bond), and *S* represents the average integral area of primary amino groups (–CH–NH bond) [11].

### 2.3. Bioprinting Model Design and Fabrication

**Figure 1:**
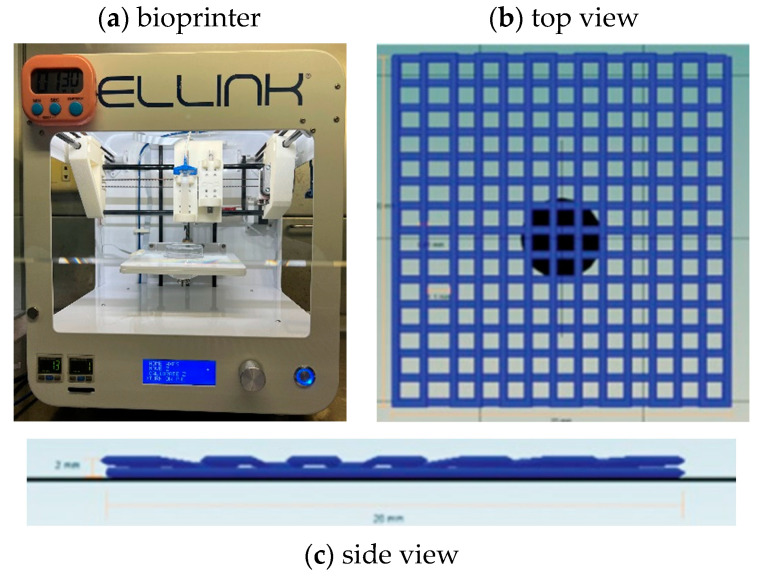
(**a**) The bioprinter used in this study. (**b**) Multilayer grid scaffold pattern top view and (**c**) side view designed by Cellink HeartWare Repetier-Host program for bioprinting of Cellink Inkredible bioprinter.

To investigate printability, we used the INKREDIBLE bioprinter (Cellink, Gothenburg, Sweden) with the Cellink HeartWare/Repetier Host Software version 2.3.2 model design to fabricate the scaffold in a square shape measuring 20 × 20 × 2 mm^3^. All samples were printed at room temperature (25 °C) and crosslinked by 365 nm wavelength UV irradiation with an intensity of 10 mW/cm^2^ for 10 min throughout the printing period; 22-gauge (22 G, inner diameter 0.644 mm) conical bioprinting nozzles (Cellink, Sweden) were fixed to 3 mL plastic cartridges for printing, as shown in Figure 1. The minimum pressure at which continuous extrusion occurs was selected, the nozzle speed was set to 5 mm/s, the infill density was set to 100%, and the distance from the needle to the print bed was optimized so that the leading edge of the flow was in line with the needle. Each printed line diameter was 0.41 mm, and the porosity size was 1.1 mm for one layer using a G-code program as a command design. Printability tests were conducted by using a glass Petri dish as the printing surface. We then imaged each print (8-megapixel camera, 1.5 µm pixel size). The ImageJ program (NIH, Bethesda, MD, USA) was used to view the threshold images and measure the printed line size.

### 2.4. Printability

The diameter measurement of the printed line evaluated the printability. The line size should be close to the designed line and nozzle diameter size. The printing accuracy was determined by comparing the printed scaffold area and the designed scaffold using ImageJ program. The percentage of printing accuracy (% accuracy) was calculated as in Equation (2):

(2) $$\%\;Accuracy=1-\frac{\left|a-b\right|}{a}\times 100$$

where: 

*a* is the area of the designed scaffold;

*b* is the area of the printed scaffold that was measured using ImageJ.

### 2.5. Mechanical Testing

Uniaxial compressive strength tests were performed on a Universal tensile testing machine (TAXT plus Stable Micro Systems, Surrey, UK) with a crosshead speed of 0.1 mm/s and a 10 N load cell. The specimen was prepared with a 20 × 20 × 20 mm^3^ size and then crosslinked by 365 nm wavelength UV irradiation with an intensity of 10 mW/cm^2^ for 20 min. 

### 2.6. Swelling Test

The swelling of the hydrogel has an impact on the quality and effectiveness of the scaffold in nutrient absorption. To remove the water content, the crosslinked GelMA 10% was crosslinked by 365 nm wavelength UV irradiation with an intensity of 10 mW/cm^2^ for 20 min before coating in thin films (n = 3) and dried at 40 °C. Subsequently, all samples were weighed and immersed in deionized water for 10 min at 37 °C. All specimens were removed from the soaking water and weighed at 15, 30, 45, and 60 min intervals of soaking time. Equation (3) was used for the calculation of the swelling ratio.

(3) $$\mathrm{Swelling}\;\mathrm{Ratio}\;(\%)=\frac{Wt-Wo}{Wo}\times 100$$

where: 

*W_t_* is the weight of the swelling hydrogel;

*W*_o_ is the weight of the dry hydrogel.

### 2.7. Cell Viability Testing

HaCaT cells, immortal keratinocyte cell lines derived from adult human skin, were cultured in Dulbecco’s modified Eagle’s medium (DMEM) supplemented with 10% fetal calf serum (Life Technologies-Gibco, Grand Island, NY, USA) and 100 mg/mL Penicillin-Streptomycin (Life Technologies-Gibco), as previously described [12]. The cells were incubated at 37 °C in a humidified environment with 5% of CO_2_. HaCaT cells were thoroughly mixed with GelMA 10% bioink in the quantity of 5 million cells per mL in a 3:1 (*v*/*v*) biological ratio of bioink to cells. The Cellink Inkredible bioprinter (Cellink, Sweden) was used to print the samples in a square form with the size of 20 × 20 × 2 mm^3^ using 22 G needles. Throughout the printing process, UV radiation with a wavelength of 365 nm and an intensity of 10 mW/cm^2^ was utilized to crosslink the scaffold. The cells’ embedded scaffold was cultivated for 24 and 72 h in a 5% CO_2_ incubator and observed using an inverted microscope with a scale bar (Olympus, Tokyo, Japan) To investigate the cell numbers in a specific area of the scaffold using the hemocytometer counting method, phase-contrast images were taken in four different locations of the scaffold, and the number of total cells (phase image) in each field area (0.1 × 0.1 mm grid) was counted by using a cell-counting tool (ImageJ NIH, Bethesda, MD, USA). Equation (4) was used to calculate the cell numbers in the scaffold.

(4) $$\mathrm{Cell}\;\mathrm{number}=\frac{X}{4}\times \left(1\times 10^{4}\right)\times \frac{bi}{m.d}\times A$$

where: 

*X* is the number of cells counted from four points on the scaffold; 

*A* is the area of the scaffold (mm^2^);

bi is the volume of printed bioink (mL);

m.d is the volume of culture media containing cells (mL).

Confocal microscopy has also been used to examine surviving cells within the scaffolds using the fluorescent dye DAPI, which binds to the cell nucleus. Confocal pictures were also captured after DAPI labeling the HaCaT cells embedded in GelMA hydrogel. The culture medium was first taken out, and then phosphate-buffered saline (PBS) was gently rinsed into the cells. At room temperature, the cells were fixed with 4% paraformaldehyde (PFA) for 10 to 15 min. The excess PFA was then washed off with PBS, enabling the DAPI dye to reach the cell nuclei; 0.1% Triton X-100 in PBS was used to permeabilize cells for 10 min. After one more PBS wash, cells were treated with 1 µg/mL of DAPI solution. Effective nucleus staining was facilitated by incubation in a 5–10 min dark environment. After that, excess DAPI was washed out of the cells with PBS in order to preserve the stained samples, and an anti-fade adhesive cover was attached to a fresh microscope slide. Consequently, a professional sealer or nail polish was applied to seal the slide. The prepared slides were then investigated under a fluorescence microscope.

### 2.8. Statistical Analysis

One-way analysis of variance (ANOVA) was carried out for the data set with more than two samples, and Duncan’s new multiple range tests were used to test for the differences between means (*p* ≤ 0.05). A paired *t*-test was used for the mean comparison of two data sets (*p* ≤ 0.05). These analyses were carried out using the SPSS Statistics software version 25.0 (IBM; Armonk, NY, USA).

## 3. Results and Discussion

### 3.1. Fish Scale GelMA Synthesis and Degree of Substitution

**Figure 2:**
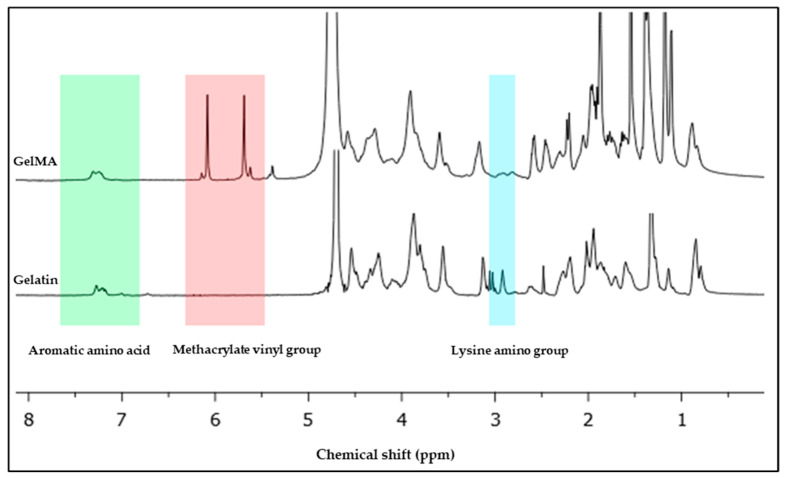
^1^H NMR spectra of the fish scale gelatin and GelMA.

The spectra of the ^1^H-NMR spectroscopy of the fish scale GelMA compared with the fish scale gelatin shown in Figure 2 exhibit the characteristic peaks of successful methacrylation from gelatin to GelMA. In particular, the observed peak around 6–7 ppm corresponds to aromatic amino acids that are naturally present in the gelatin backbone. The presence of the methacrylate vinyl groups confirmed this through observed peaks at approximately 5.3 and 5.7 ppm, which confirmed the successful synthesis. The lysine resonance of approximately 2.8–3.0 ppm is characteristic of side chain protons determined by aggregation analysis [13]. This was determined by analyzing the peak of the vinyl methacrylate group and comparing it with the peak of the amino acid lysine that is present in the gelatin structure. The degree of substitution (DS) calculated from Equation (1) is approximately 96.38%.

The NMR spectra provided evidence of GelMA synthesis. Peaks in the vinyl methacrylate group show methacrylate substitution and a drop in the lysine peak that showed up on the gelatin structure. This confirms that gelatin obtained from fish scales can successfully synthesize gelatin methacrylate [14]. The aromatic amino acid peaks determine the inherent structure of gelatin, which is derived mainly from the amino acid sequences of collagen. It remains intact mainly after methacrylation. This is important because maintaining gelatin’s natural biological signals is essential for biocompatibility and cell interaction [15]. The high DS value indicates that the necessary substitution of amino groups of gelatins by the methacryloyl groups that provide GelMA exhibits a large number of crosslinking sites, which is critical for achieving a stable hydrogel. However, striking a balance is important. Although higher DS produces stronger hydrogel, it also affects cell survival because each type of cell requires a different hydrogel stiffness. The NMR results suggest that fish scales can be efficient and sustainable sources of GelMA synthesis. The resulting GelMA structure appears similar to that obtained from mammalian sources in a previous study that showed the structure of GelMA from porcine [16].

### 3.2. Printability

**Figure 3:**
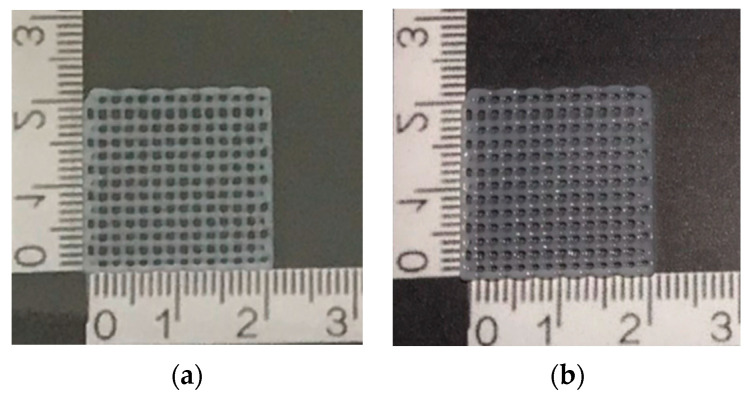
Demonstrated scaffolds from GelMa fish scales using 3D bioprinting technique: (**a**) 10% GelMA and (**b**) 15% GelMA.

**Figure 4:**
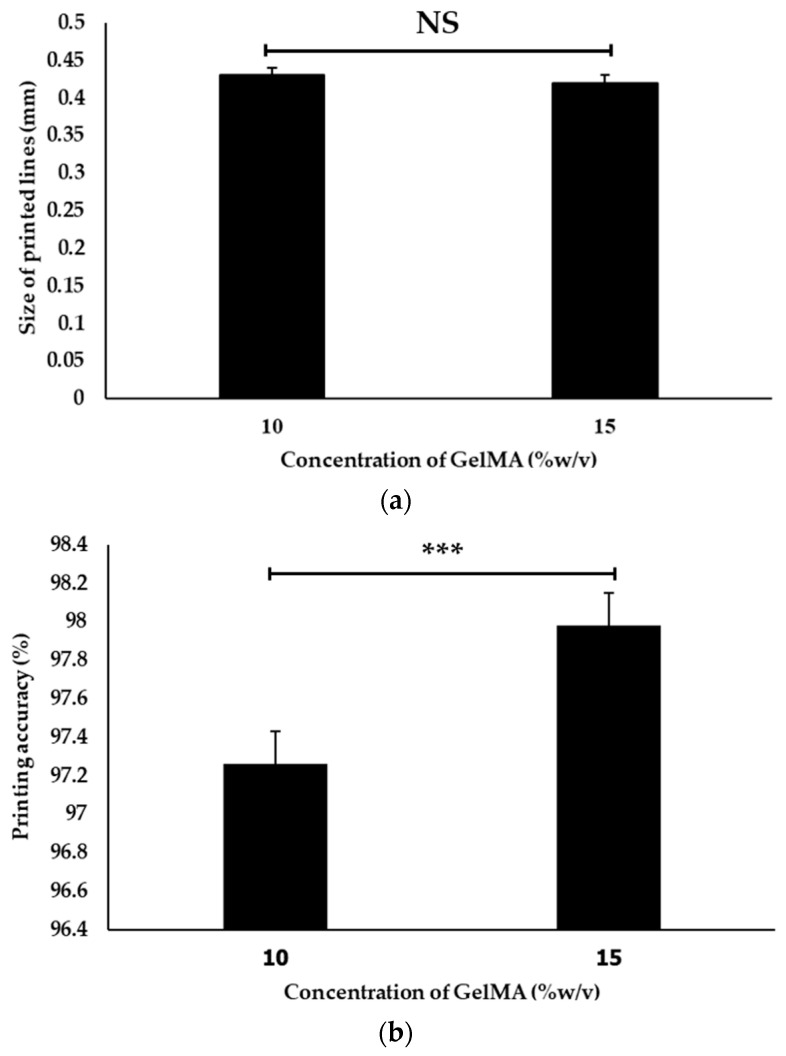
The size of the printed lines (**a**) and the printing accuracy of 10% GelMA and 15% GelMA (**b**) (n = 3, *** is significant difference, and NS is non-significant).

Figure 3 and Figure 4 show the effect of two concentrations of fish scale GelMA on the size of the printed lines, in which the printing accuracy of both concentrations, 10% and 15% (*w*/*v*), were compared. It was observed that at a 10% concentration, the printed line size was approximately 0.43 ± 0.01 mm, while at a 15% concentration, the size decreased to approximately 0.42 ± 0.01 mm. The calculation of the printing accuracy from Equation (2) was approximately 97.26 ± 0.16% and 97.98 ± 0.17% at concentrations of 10% and 15%, respectively. Thus, there was a significant difference between the printing accuracy and no significant difference between the sizes of the printed lines at these two concentrations.

The above data show that increasing the concentration of fish scale GelMA from 10% to 15% (*w*/*v*) resulted in a slight decrease in the printed line size. However, this decrease was not statistically significant (*p* > 0.05). To reduce the raw material employed for the fabrication, 10% GelMA was selected to further investigate the compressive property, swelling, and cell proliferation. The printability of fish scale GelMA may not be sensitive to the changes in concentration within the tested range. This may be useful for applications where it is difficult to maintain an accurate concentration of the specimen. A slight deviation did not significantly affect the printing ability. However, other factors may play different roles, such as the printing speed [17], nozzle diameter [18], pressure [19], temperature [20], inherent properties of fish scale GelMA [21], etc.

### 3.3. Compression Behavior

**Figure 5:**
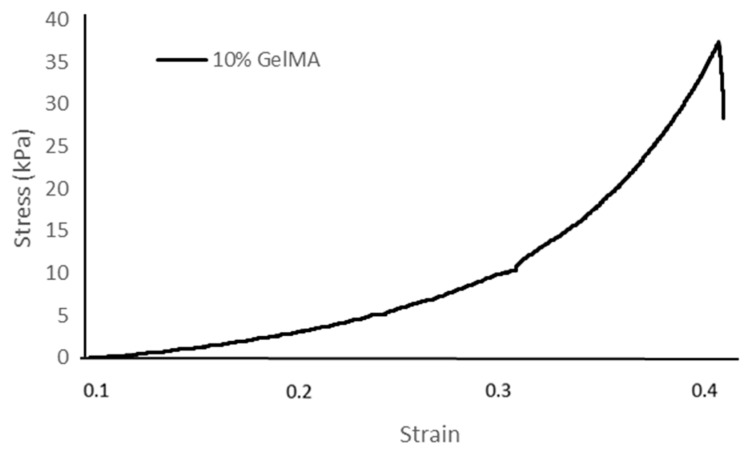
Stress–strain curve of the 10% GelMA hydrogel.

Table 1 and Figure 5 show the stress–strain relationship of fish scale GelMA in 10% GelMA concentrations (*w*/*v*) under applied compressive stress. The compressive property of fish scale GelMA exhibits a steep increase, especially after a strain of approximately 0.3. At higher strains, around 0.4, the 10% GelMA sample can reach a stress of about 37 kPa. In addition, other factors affect the strength, such as the degree of substitution [22], the completeness of crosslinking [23,24] that consists of the amount of photoinitiator, a 365 nm UV light intensity, and the crosslinking time. However, all of the factors mentioned above can negatively impact the cell viability, so great care must be taken when adjusting the various parameters [25].

### 3.4. Swelling Property

**Figure 6:**
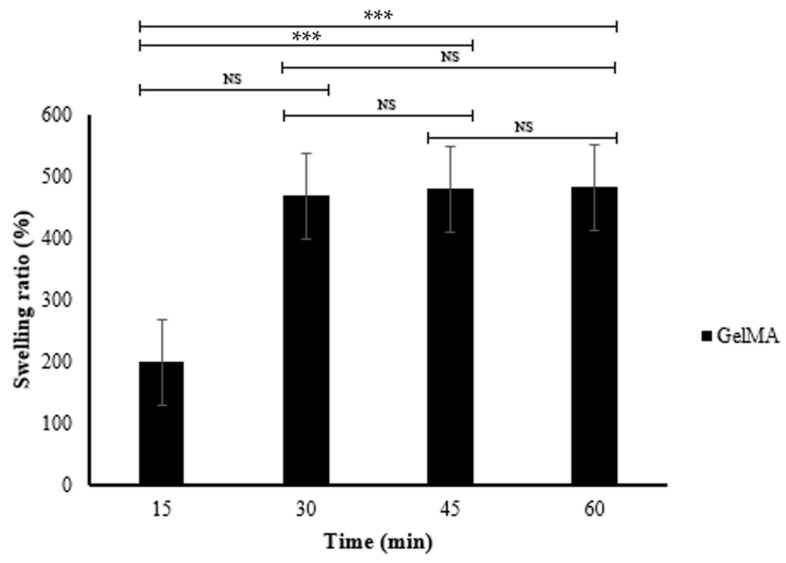
The swelling ratio of the fish scale GelMA (n = 3, *** is significant difference, and NS is non-significant).

Table 2 and Figure 6 show the swelling behavior of the fish scale GelMA bioink over 60 min. The table presents the dry weight, swelling weight, and swelling ratio (%) of various time periods. At 15 min, GelMA had a swelling ratio of 199.30 ± 16.21%. This ratio drastically increased to 468.49 ± 17.36% within 30 min. After that, the swelling ratio was decelerated from 478.89 ± 34.19 at 45 min to 482.12% at 60 min.

The dramatic increase in the swelling ratio from 199.30 ± 16.21% to 468.49 ± 17.36% in the first 30 min indicates the rapid swelling ability of the GelMA bioink. This may be due to the inherent porosity, which promotes water absorption. After 30 min, the swelling ratio is significantly reduced. The differences between the 30, 45, and 60 min time periods were not statistically significant; this distance may indicate that the GelMA bioink almost reached equilibrium, where the absorption capacity of the fluids was decreased. The initial scaffold porosity is critical for the penetration of cell nutrient fluids [26], and the subsequent stability ensures the integrity of the hydrogel structure. The factors that influence the swelling behavior are the crosslink density [27], pH [28], and the external environments.

### 3.5. Cell Viability

**Figure 7:**
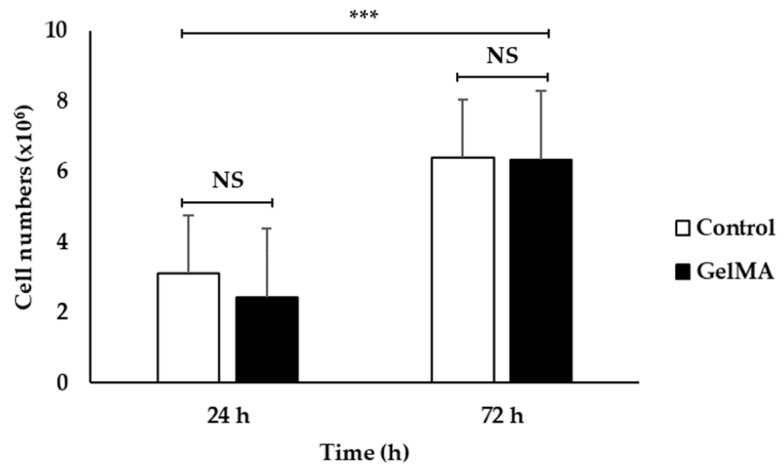
The cell viability of embedded HaCaT cells in fish scale GelMA scaffold compared to the control HaCaT cells at the cultivation time of 24 and 72 h (n = 3, *** is significant difference, and NS is non-significant).

**Figure 8:**
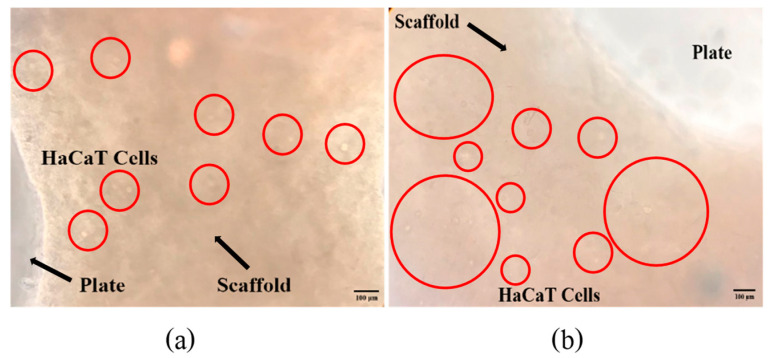
The survival of HaCaT cells inside the GelMA bioink scaffolds after incubation times of 24 h (**a**) and 72 h (**b**).

**Figure 9:**
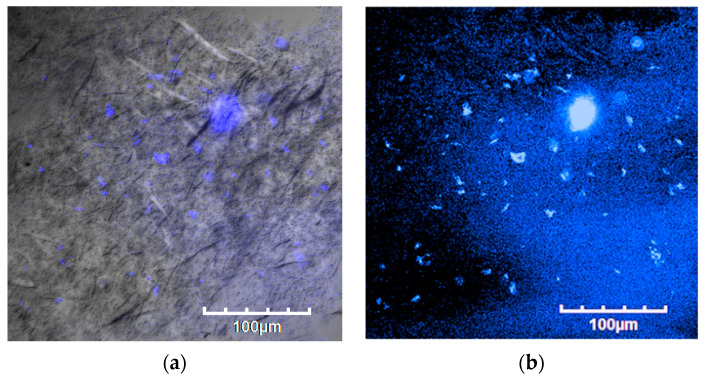
Laser-scanning confocal microscope images of HaCaT cells embedded in GelMA bioink scaffolds after an incubation time of 72 h. (**a**) Bright-field photograph of fluorescent DAPI-stained cells and (**b**) dark-field photograph of fluorescent DAPI-stained cells.

Figure 7 shows a bar graph of cell numbers that survive inside the fish scale GelMA scaffold compared to the control group on the plate within 72 h. At 24 h, both the control and GelMA samples show similar numbers of survival cells (*p* > 0.05). Similarly, at 72 h, the number of alive cells surviving inside the scaffolds from fish scale GelMA and the control group was significantly increased compared to at 24 h. Figure 8 presents the survival of HaCaT cells inside the scaffolds. The translucent white spheres circled in red represent HaCaT cells after 24 and 72 h of culture investigated under a microscope. These data confirmed the results from confocal microscopy. The captured images of cells inside the GelMA matrix of fish scales labeled with DAPI nuclei are shown in Figure 9. HaCaT cells were distributed throughout the matrix, as indicated by the blue fluorescent spots. However, the observed large blue stop might be aggregated cell clusters.

The results above show that using GelMA made from fish scales can increase the number of survival-embedded HaCaT cells from 24 to 72 h. They could be seen by the naked eye in the confocal microscope pictures (red circles), and by the DAPI staining, which shows many fluorescent cell nuclei. This means that the GelMA made from fish scales in this study could be a suitable matrix for cell survival and growth. If the culture continues, the individual cells will aggregate until they can grow into tissues. The control group cultured cells in 2D on a well plate, which had limitations in growth because when the cells grew an entire area, the cells would die due to the limited area for the cells to proliferate. In contrast, a 3D cell culture scaffold can promote longer-term cell growth [21]. Consistent with the present finding, previous research that used gelatin from the same fish scales mixed with alginate found that cells were able to survive inside the cell culture scaffold [21].

### 3.6. Comparison of the Critical Properties between Two Different Gelatin Sources

The comparison of gelatin sources of GelMA bioink from commercial porcine and derived fish scales was investigated to ensure the printability and mechano-biological properties of fish scale GelMA. The printing accuracy, compressive strength, and cell viability of both GelMA bioink are presented in Table 3. The printing accuracy of GelMA bioink from commercial porcine gelatin and fish scale gelatin were 98 and 97%, respectively, which was not significantly different (*p* > 0.05) [29]. The compressive strength of GelMA bioink from the porcine gelatin was higher than that of the GelMA bioink from fish scale gelatin resources [7]. Despite this, the GelMA bioink from fish scale gelatin exhibited non-cytotoxicity, which indicated cytocompatibility. Similarly, work by others also reported the application of porcine GelMA in tissue engineering [7,29]. This finding suggests that the newly invented biomaterial has potential to be used in tissue engineering applications [30,31].

## 4. Conclusions

This study found that the synthesized fish scale GelMA has suitable properties as a bioprinting ink for fabricating cell culture scaffolds using 3D bioprinting techniques. The material has a printing accuracy of up to 97%. The fish scale GelMA can withstand the compressive pressure of 85 kPa, swells up to 482%, and is cytocompatible after testing with HaCaT cells. It was also observed to have properties and performance similar to commercial GelMA. This newly invented GelMA is environmentally sustainable and made from fish scales, which are typically discarded as waste. As a result, this study can serve to promote a paradigm shift toward environmentally conscious manufacturing and religious constraints in clinical practice.

## Figures and Tables

**Table 1:** The GelMA test results for average mechanical properties at a concentration of 10%.

Compressive Strength (kPa)	Ultimate Strength (kPa)	Ultimate Strain (%)
84.8	37.3	44.0

**Table 2:** The swelling ratio of the fish scale GelMA.

Bioink	Dry Weight (g)	Swelling Weight (g)	Time (min)	Swelling Ratio (%)
GelMA	0.31 ± 0.02	0.93 ± 0.11 ^b^	15	199.30 ± 16.21 ^b^
	0.31 ± 0.02	1.76 ± 0.06 ^a^	30	468.49 ± 17.36 ^a^
	0.31 ± 0.02	1.79 ± 0.01 ^a^	45	478.89 ± 34.19 ^a^
	0.31 ± 0.02	1.80 ± 0.01 ^a^	60	482.12 ± 34.40 ^a^

Note: Data are presented as mean ± SD. Different superscripts in the same column indicate statistical differences (*p* ≤ 0.05).

**Table 3:** Comparison of the critical properties of the 10% GelMA bioink for bioprinting by using fish scale gelatin compared to commercial porcine gelatin.

Properties	Type of GelMA Bioink from Different Gelatin Sources	
	Commercial Porcine Gelatin ^a^	Fish Scale Gelatin
Printing accuracy (%)	98	97
Compressive strength (kPa)	100	85
Non-cytotoxicity	✓	✓

^a^ The experimental values are from [7,22]. ✓ is non-cytotoxicity.

## Data Availability

The datasets used and/or analysed during the current study are available upon reasonable request.
